# Overview of glutamine metabolism in stromal components of the tumor microenvironment and potential anti-tumor therapies

**DOI:** 10.1016/j.gendis.2025.101834

**Published:** 2025-08-26

**Authors:** Zizhuo Li, Jiapeng Deng, Hai Wang, Tao Liu, Yuyang Zhou, Pei Ouyang, Xuan Liang, Xian Zhang, Songtao Qi, Yaomin Li

**Affiliations:** a Department of Neurosurgery, Institute of Brain Disease, Nanfang Hospital, Southern Medical University, Guangzhou, Guangdong 510515, China; b The First School of Clinical Medicine, Southern Medical University, Guangzhou, Guangdong 510515, China

**Keywords:** Anti-tumor therapy, Glutamine, Glutamine metabolism, Stromal cells, Tumor microenvironment, Tumor stromal components

## Abstract

As a critical metabolite in the tumor microenvironment, glutamine plays a crucial role in tumor progression, and its dual effects on promoting and inhibiting tumors have garnered increasing attention in recent years. Glutamine metabolism in tumor cells has been extensively studied; however, there is currently a lack of a comprehensive description of how it interacts with tumor stromal components in the tumor microenvironment. This review focuses on the interaction of glutamine metabolism and a range of tumor stromal components, such as macrophages, dendritic cells, T cells, fibroblasts, collagen, and blood vessels in the tumor microenvironment, as well as a summary of current prospective anti-tumor therapeutics targeting glutamine metabolism. Furthermore, this study discusses the shortcomings of mechanism research, metabolic complexity, and metabolic therapy for glutamine metabolism and proposes future research directions that are expected to provide a theoretical foundation for clinical cancer treatment strategies.

## Graphical abstract

**Figure:**
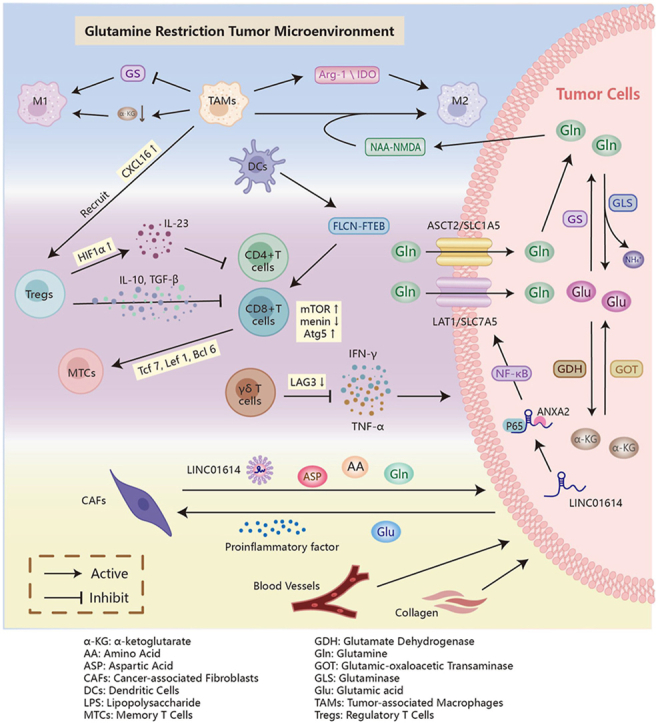

## Introduction

Glutamine (Gln), a vital nutrient for tumor cells and stromal cells in the tumor microenvironment (TME), plays a crucial role in tumorigenesis and progression.^1^ Researchers are intrigued by the intimate link between Gln as a tumor-promoting agent and tumor cell proliferation, metastasis, and invasion. Gln in cancer cells not only increases ammonia generation, which promotes stromal cell autophagy, but also boosts mitochondrial tricarboxylic acid (TCA) cycle and oxidative phosphorylation, resulting in increased ATP production, cancer cell biomass and proliferation.^2^ In contrast, Gln inhibition promotes tumor cell death and slows tumor development. Inhibition of tumor suppressor sirtuin 4 (SIRT4)-mediated Gln anaplerosis is required for successful cell cycle arrest produced by the DNA damage response.^3^ Autophagy-related 12 (ATG12) plays a vital function in regulating L-Gln homeostasis. However, ATG12 deletion lowers intracellular L-Gln levels and promotes cell lethality upon L-Gln depletion, impeding the evolution of head and neck squamous cell carcinoma.^4^ Currently, there exist reviews that focus on the phenomenon of Gln addiction in tumor cells and outline the regulation and effects of Gln metabolism in tumor cells.^5^

Furthermore, the anti-tumor activity of Gln has been gradually investigated. Oral Gln supplementation may increase natural killer (NK) cells activity via the glutathione/interleukin-2 (IL-2) pathway, activating NK cells that participate in anti-tumor immunity by producing cytokines such as interferon.^6^ Oral Gln supplementation increases tumor necrosis factor-alpha (TNF-α) production by macrophages, IL-1 and IL-6 secretion, and chemotoxicity of monocytes and neutrophils, resulting in anti-tumor effects through humoral and cellular immunity.^7^ In addition, oral Gln dramatically increased the expression of pro-apoptotic factor Bad while inhibiting the expression of B-cell lymphoma 2 (Bcl-2). Meanwhile, Gln boosted the activity of calpain I, caspase-8, caspase-9, and poly(ADP-ribose) polymerase (PARP), activating the apoptotic pathway and inhibiting tumor growth in gastric cancer tumor-bearing mice.^7^ The findings seem to suggest that exogenous Gln supplementation may improve anti-tumor immunity via altering glutathione levels in the circulation rather than tumor tissues, triggering apoptotic pathways, and boosting immune cells. As a result, the key to solving the problem is to manage Gln metabolism so that its anti-tumor effect outweighs its pro-tumor effect.

In summary, Gln metabolism is critical in the process of tumorigenesis and development, and the Gln metabolism of tumor cells has received extensive attention. The TME, as a fertile soil for tumor growth, differs significantly in its Gln-deficient metabolic environment compared with normal tissues. Stromal cells in the TME, including non-tumor cells, such as macrophages, dendritic cells (DCs), T cells, and fibroblasts, as well as mesenchymal components such as collagen and blood vessels, play a crucial role in shaping and adapting to the unique metabolic environment. Stromal cells in the TME and tumor mesenchymal components are together referred to as tumor stromal components. There is currently a lack of review on how Gln metabolism interacts with tumor stromal components in the TME. Therefore, the following will focus on the Gln metabolism process and its effects on tumor stromal components within the TME, and on this basis, summarize potential recent tumor treatment methods targeting Gln metabolism, as well as future research directions in the field of Gln metabolism, which are expected to provide a theoretical basis for clinical cancer treatment strategies.

## Glutamine metabolism pathway

**Figure 1:**
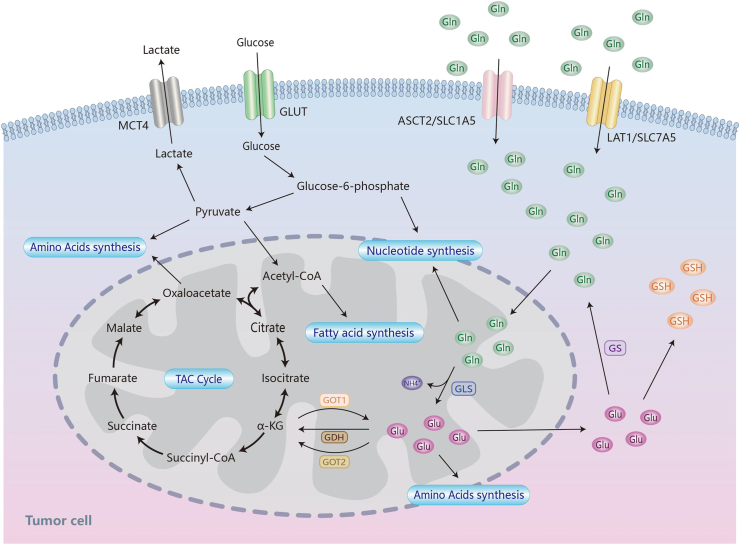
The glutamine metabolic pathway. Cells absorb glutamine from the matrix via the glutamine transporters SLC1A5 and SLC7A5, where GLS catalyzes the generation of Glu and NH4^+^ in the mitochondria. GDH catalyzes the conversion of Glu to α-KG, which enters the TCA cycle to support cellular energy and serves as an intermediate in amino acid and lipid synthesis. GOT1 and GOT2 facilitate the conversion between glutamate and α-KG. Furthermore, glutamate contributes to the formation of GSH, supporting cellular stability. Together with glucose metabolism, lipid metabolism, amino acid synthesis, and nucleotide synthesis, the glutamine metabolism pathway forms a tumor metabolic network, which is essential for tumor progression. GLS, glutaminase; GOT, glutamic-oxaloacetic transaminase; GSH, glutathione; GDH, glutamate dehydrogenase; Glu, Glutamic acid; Gln, glutamine; TCA, tricarboxylic acid; GLUT, glucose transporter; α-KG, α-ketoglutarate.

Gln has a high metabolic level to meet the tumor cell proliferation and expansion requirements. Gln enters cells via transporters such as alanine-serine-cysteine transporter 2 (ASCT2, also termed SLC1A5) and solute carrier family 7 member 5 (SLC7A5), where it is converted into glutamate and ammonium ions via glutaminase (GLS) catalysis. The expression of Gln transporter SLC1A5 in tumor cells is regulated by a number of genes and tumor factors, and ASCT2-dependent Gln uptake can enhance tumor cell proliferation, while inhibiting cancer cell death and autophagy, resulting in tumor development.^8^ GLS, commonly referred to as GLS1, is up-regulated to increase Gln breakdown and promote tumor growth.^9^ Unlike GLS1, GLS2 appears to play a tumor suppressive role. GLS2 is a target of p53 and a crucial enzyme that converts Gln to glutamate. It is also intimately related to glutathione synthesis. Overexpression of GLS2 inhibits tumor cell proliferation and colony formation.^10^ GLS2 promotes ferroptosis, which suppresses tumors by increasing the generation of lipid reactive oxygen species through the conversion of glutamate to α-ketoglutarate.^11^ Glutamic acid is converted to α-ketoglutarate in the mitochondria through oxidative deamination by glutamate dehydrogenase or transamination. It then enters the TCA cycle, which provides energy and intermediates for amino acid and lipid synthesis. When glucose is depleted, glutamate dehydrogenase becomes the predominant channel for supplying Gln carbon to the TCA cycle, which is required for cell viability. Gln-associated cysteine synthesis of glutathione from glutamic acid, cysteine, and glycine to keep cells stable. Furthermore, Gln plays a role in the production of several non-essential amino acids.^5^^,^^12^ Glutamic-oxaloacetic transaminase (GOT) indirectly affects Gln metabolism but is not directly engaged. GOT is typically found in both the cytosolic form GOT1 and the mitochondrial form GOT2.^13^ GOT2 catalyzes the conversion of Glu and oxaloacetate into α-ketoglutarate and aspartic acid, whereas GOT1 catalyzes the opposite reaction.^14^ Gln synthetase, also known as glutamate-ammonia ligase (GLUL), is the sole enzyme capable of producing Gln on its own, and it is highly expressed in the TME, supplying Gln to cancer cells and playing a crucial part in the metabolic process of tumor growth.^15^ Gln synthetase-mediated Gln synthesis has been associated with rapid cancer cell proliferation in various malignancies, such as glioblastoma and hepatocellular carcinoma. It could potentially have a function in TCA cycle activity and nitrogen production^16^^,^^17^ (Fig. 1).

Gln metabolism is closely associated with multiple metabolic processes, such as glycolysis and lipid metabolism, forming a complex metabolic network within the TME that supplies intermediates for various pathways.^18^ Within the TME, alterations in oxygen availability and nutrient deficiencies shift the primary source of citric acid/isocitrate in the TCA cycle toward the carboxylation of Gln-derived α-ketoglutarate, leading to the production of acetyl-CoA and oxaloacetate.^19^ In the cytoplasm, citric acid is broken down into oxaloacetate and acetyl-CoA through an ATP-citrate lyase-mediated mechanism.^20^ The excess acetyl-CoA facilitates fatty acid synthesis, whereas oxaloacetate is converted into pyruvate.^20^ Moreover, Gln catabolism can result in the conversion of malic acid, an intermediate in the TCA cycle, into pyruvate, which is then processed by pyruvate dehydrogenase to generate acetyl-CoA, serving as a carbon source for fatty acid synthesis^21^ (Fig. 1).

## Glutamine affects the function of immune cells in the TME

Immune cells are an essential component of the TME, and immune cell malfunction plays a significant role in tumor formation. Gln is an essential fuel source for immune cells in the TME, and its metabolism is tightly linked to nitrogen metabolism, providing most of the nitrogen necessary for nucleic acid and amino acid synthesis.^22^ The impact of Gln metabolism on the function of T cells, macrophages, and DCs will be discussed here.

### Glutamine metabolism in T cells

Gln is a key nutrient for T cells, and adequate Gln metabolism is critical for maintaining T cell homeostasis and boosting T cell anti-tumor immunity. Several investigations have found that Gln is an essential nutrient that regulates T cell immunological activity and effector functions.^23^^,^^24^ In activated T cells, Myc-mediated transcription of SLC32a1, SLC32a2, and GLS2 regulates Gln uptake and metabolism, which is closely related to the manufacture of polyamines required for T cell proliferation.^25^

**Figure 2:**
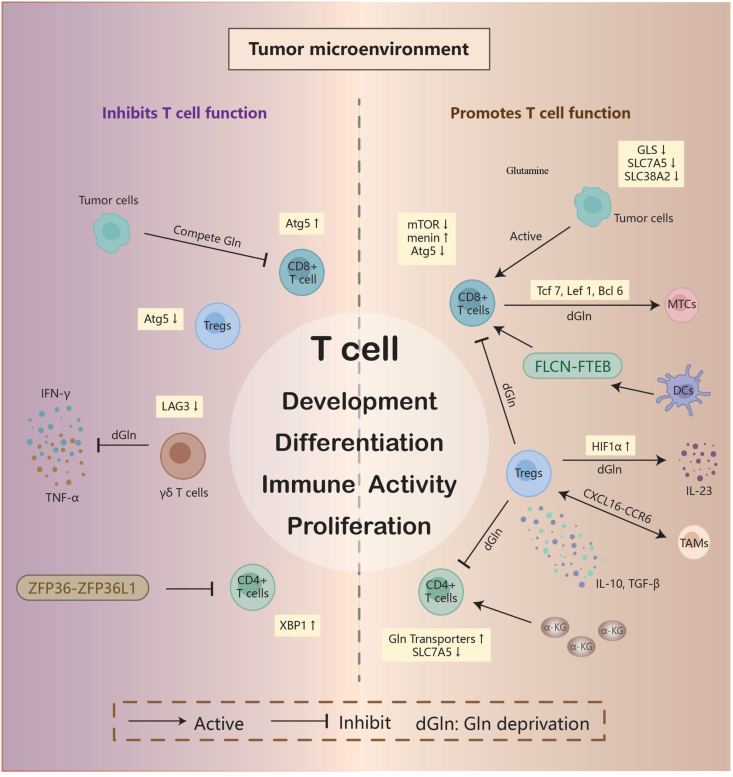
Immune effects mediated by T cell subsets in the tumor microenvironment. i) CD8 T cells: Glutamine modulates DC antigen presentation via the FLCN-TFEB axis, activating CD8^+^ T cells and inhibiting tumor development. Menin helps to sustain T cell function by decreasing mTORC1 activity and, as a result, glutamine metabolism. Glutamine restriction increases gene expression of the transcription factors Tcf7, Lef1, and Bcl6, accelerates the development of CD8^+^ T cells into memory cells, and enhances CD8^+^ T cell anti-tumor activity. Tumor cells compete for glutamine in the tumor microenvironment, inhibiting tumor-infiltrating T lymphocyte activity; however, down-regulation of SLC38A2, GLS, and SLC7A5 in tumor cells destroys glutamine metabolism, restoring effective recruitment and immune activation of antigen-specific anti-tumor CD8^+^ T lymphocytes. Deletion of the autophagy gene ATG5 reduces autophagy and raises glutamine levels, promoting inflammatory T cells and having anti-tumor immunological actions. ii) CD4^+^ T: Down-regulation of SLC7A5 increases CD4^+^ T cell infiltration while delaying tumor growth and immunosuppression. Overexpressing the glutamate transporter or supplementing with the glutamine-derived TCA cycle intermediate α-KG helps maintain cellular stability. ZFP36 and ZFP36L1 work directly to limit CD4^+^ T cell growth. Furthermore, XBP1 activation reduces glutamine absorption by CD4^+^ T cells, which promotes tumor development. iii) Tregs: Glutamine deprivation activates HIF1a, leading to increased IL-23 production, Treg proliferation and activation, increased TGFβ and IL-10 expression, and improved Treg suppression of CD4^+^ and CD8^+^ T cell proliferation. A subgroup of TAMs with high glutamine synthetase levels may interact with Tregs to establish an immunosuppressive environment through CXCL16-CCR6 signaling. Additionally, deletion of the autophagy gene ATG5 raises glutamine levels, suppresses Treg production, and has anti-tumor immune properties. iv) γδT cells: Glutamine restriction in the microenvironment down-regulates LAG3 expression in γδT cells, lowering the amounts of key effector cytokines like IFN-γ and TNF-α. This promotes the creation of an immunosuppressive environment. Treg, regulatory T cell; MTC, memory T cell; TAM, tumor-associated macrophage; DC, dendritic cell; Gln, glutamine; α-KG, α-ketoglutarate.

On the one hand, Gln metabolism regulates T cell-mediated immunological responses. Down-regulating SLC7A5, a critical enzyme in Gln metabolism, led to enhanced T cell infiltration and decreased tumor development and immunosuppression in triple-negative breast cancer.^26^ Gln regulates mammalian target of rapamycin (mTOR) activation and O-glcNAc glycosylation in effector T cells to promote proper T cell growth and function.^27^ In triple-negative breast cancer, tumor cells compete for Gln in the TME, suppressing tumor-infiltrating T cell activity, whereas defective Gln metabolism by tumor cells restored the immunoreactivity of antigen-specific anti-tumor CD8^+^ T cells in a GLS-deficient animal model.^28^ Menin, a tumor suppressor, prevents effector CD8^+^ T cell failure by inhibiting mTORC1 activity and, as a result, various cellular metabolisms, including Gln metabolism, sustain T cell function.^29^ In contrast, in menin-deficient CD8^+^ T cells, mTORC1 signaling promotes Gln metabolism, which causes histone H3K27me2/me3 demethylation and T cell dysfunction.^29^ Inhibition of Gln metabolism in T cells and cancer cells in the TME may contribute to increased proliferation of CD8^+^ T cell subsets.^30^ This could be related to effector T cells' ability to respond to Gln blockage by increasing alternate metabolic pathways, such as pyruvate carboxylase and acetate metabolism.^31^ As one interesting comment put it, the loss of tumor cells is the acquisition of T cells.^32^ Gln restriction increased gene expression of transcription factors, including transcription factor 7 (Tcf7), lymphoid enhancer binding factor 1 (Lef1), and B-cell lymphoma 6 (Bcl6), and enhanced T-cell differentiation of tumor-specific CD8^+^ T cells toward long-lived memory precursor cells after T cell receptor-stimulated activation. This resulted in increased production of interferon-gamma (IFN-γ) and TNF-α when encountering tumor cells.^33^ Differentiation into memory cells improves CD8^+^ T cell anti-tumor activity without impairing effector functions.^34^, ^35^, ^36^, ^37^ Autophagy reduces Gln levels in tumors and decreases cytotoxic T cell activity, which promotes the growth of malignant salivary gland tumors.^38^ In contrast, loss of the critical autophagy gene autophagy-related 5 (ATG5) results in elevated Gln levels in the TME, which boost inflammatory T-cells and suppress regulatory T cell (Treg) formation to provide anti-tumor immunological effects.^38^ Studies have demonstrated that in glucose shortage, X-box binding protein 1 (XBP1), a key factor in malignancy, is activated to limit Gln uptake in CD4^+^ T cells via modulating Gln carrier abundance. In contrast, overexpressing Gln transporters or supplementing with Gln-derived TCA cycle intermediates like α-ketoglutarate restores mitochondrial respiration in T cells.^39^ Zinc finger protein 36 (ZFP36) and zinc finger protein 36-like 1 (ZFP36L1) directly inhibit transcript stability and/or ribosome binding, hence restricting Gln anaplerosis and CD4^+^ T cell differentiation.^40^ Despite abundant evidence showing that the control of enzymes involved in Gln metabolism plays a critical role in immune cell effector function, there is still a knowledge gap in this sector. SLC38A1 and SLC38A2, two key amino acid transporters, are up-regulated after T cell receptor-mediated activation,^24^^,^^41^ although their significance in T cell growth and function remains unknown^28^ (Fig. 2).

On the other hand, Gln metabolism is involved in shaping the immunosuppressive microenvironment. In Gln-addicted clear cell renal cell carcinoma, macrophage-derived IL-23 increased Treg proliferation and activation, increased the expression of Treg-derived transforming growth factor-β (TGF-β) and IL-10, and enhanced Treg-dependent suppression of responder CD4^+^ and CD8^+^ T cell proliferation, thereby enhancing the immunosuppressive function of Tregs.^42^ In the Gln-deficient TME, the expression of the Gln transporter SLC1A5 and the genes ornithine aminotransferase (OAT) and GLS in γδ T cells that directly regulate Gln metabolism was considerably up-regulated.^43^ Furthermore, Gln deficiency increased expression of the immunosuppressive gene lymphocyte-activating 3 (LAG3).^43^ *In vitro* studies have demonstrated that Gln metabolism is enhanced in hepatocellular carcinoma-infiltrated γδ T cells. Extracellular Gln deprivation decreases levels of important effector cytokines in γδ T cells, such as IFN-γ and TNF-α, which promote the creation of an immunosuppressive microenvironment^43^ (Fig. 2).

### Glutamine metabolism in myeloid cells

#### Macrophages

A Gln-deficient milieu or insufficient Gln uptake might cause macrophages to produce cytokines, resulting in the establishment of a Treg-mediated immunosuppressive microenvironment. Gln depletion by clear cell renal cell carcinoma cells causes a local Gln deficiency, inducing tumor-infiltrating macrophages to release IL-23 through activation of hypoxia-inducible factor 1α (HIF1α). IL-23 stimulates Treg proliferation, causes immunosuppressive function, and increases the production of IL-10 and TGF-β, limiting tumor cell death by cytotoxic lymphocytes.^44^ The tumor-associated macrophage (TAM) subpopulation APOE + CTSZ + TAM (Macro_APOE/CTSZ), which expresses Gln synthetase, may interact with Tregs via C-X-C motif ligand 16 (CXCL16)-C-C motif chemokine receptor 6 (CCR6) signaling to generate an immunosuppressive microenvironment.^45^ The transcriptional repressor homeobox containing 1 (HMBOX1) reduced intracellular Gln levels by down-regulating the Gln transporter SLC1A5, which inhibited macrophage proliferation. In contrast, overexpression of SLC1A5 reversed HMBOX1's inhibitory effect on macrophage proliferation^46^ (Fig. 2).

**Figure 3:**
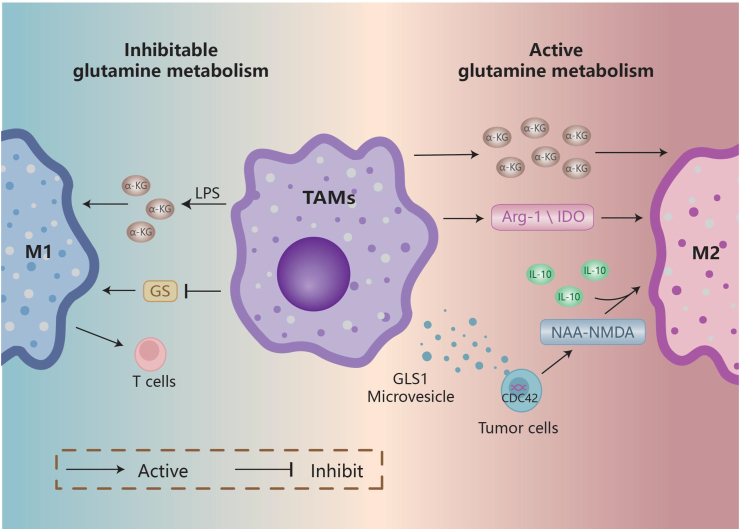
Glutamine metabolism influences TAM differentiation. Improving glutamine metabolism promotes M2 TAM polarization, which supports tumor growth. On the other hand, down-regulating glutamine metabolism promotes M1 TAM polarization, which destroys the immunosuppressive microenvironment and stops tumor growth. When combined with IL-10, glutamine addiction has been shown to make cancer cells make more NAA, block NMDA receptors, and promote M2-like tumorigenic differentiation. In tumor cells, CDC42-mediated GLS1 microvesicle release increases glutamine metabolism and macrophage M2-polarization. TAMs increased Arg-1 and IDO expression, improved glutamine synthesis and catabolism, and promoted TAM polarization to the M2 type. Glutamine lysis produces α-KG, which plays an important role in inducing the M2 phenotype of macrophages. However, glutamate metabolism inhibition negatively affects this process and reinforces the pro-inflammatory phenotype of classically activated (M1) macrophages. Furthermore, TAMs with low glutamine levels demonstrated a higher propensity for M1 phenotypic polarization in response to LPS stimulation than TAMs activated under glutamine-adequate circumstances. GS inhibition changes M2-polarized macrophages to M1 type, which makes it easier for T cells to attach and lessens their ability to suppress T cells. GS, glutamine synthetase; LPS: lipopolysaccharide; NAA, N-acetylaspartate; NMDA, N-methyl-d-aspartate; TAM, tumor-associated macrophage; α-KG, α-ketoglutarate.

Enhanced Gln metabolism promotes the polarization of TAMs to the M2 type and has pro-tumorigenic effects, while inhibiting Gln metabolism causes TAMs to polarize to the M1 type, disrupts the immunosuppressive microenvironment, and limits tumor growth. Gln addiction causes cancer cells to secrete N-acetylaspartate, inhibits N-methyl-d-aspartate receptors, and boosts Gln synthetase expression in macrophages with IL-10, creating an M2-like tumorigenic phenotype in TAMs.^47^ TAMs up-regulate the production of arginase-1 (Arg-1) and indoleamine 2,3-dioxygenase (IDO) (an enzyme that causes tryptophan depletion), which increases Gln synthesis and catabolism, encouraging TAM polarization toward M2 TAMs and generating an immunosuppressive microenvironment.^48^ Tumor cells stimulate macrophage Gln metabolism, M2 macrophage polarization, and macrophage pro-angiogenic activity via CDC42-mediated GLS1 microvesicle release, resulting in trastuzumab resistance in epidermal growth factor receptor 2 (HER2)-positive gastric cancers.^49^ The synthesis of α-ketoglutarate by Gln catabolism is necessary for M2-type activation of macrophages, while a low α-ketoglutarate/succinate ratio maintains M1 macrophages and alters the macrophage anti-tumor immune response.^50^ Gln synthetase inhibition shifted M2-polarized macrophages to an M1 phenotype, increased macrophage ability to trigger T-cell recruitment, decreased T-cell suppressive potential, and impaired endothelial cell branching and cancer cell motility^51^ (Fig. 3).

TAMs also employ glutamyl metabolism to supply energy to themselves and other cells in the TME. In glioblastoma, TAM exposure to tumor cells up-regulates gene expression of glutamate ionotropic receptor AMPA type subunit 2 (GRIA2, also termed GluA2 or AMPA receptor 2), SLC1A2 (also termed EAAT2), SLC1A3 (also termed EAAT1), and Gln synthetase, partially utilizes glutamate and Gln to meet their own energy needs, and provides energy to glioma cells and astrocytes through Gln release.^52^

#### Dendritic cells

DCs, the key antigen-presenting cells in the TME, are not affected by Gln depletion because their surface markers and secreted cytokines are unaffected, and DCs indirectly activate effector T cells via the Gln metabolism pathway, thus producing anti-tumor effects. Although the quantity and function of DCs have decreased in cancer patients, they remain a vital component in the transport of tumor antigens, coordinating innate and adaptive immune responses, as important antigen-presenting cells in the TME.^53^ Gln deprivation does not affect the expression of DC surface markers CD1a, dendritic cell-specific ICAM-grabbing non-integrin (DC-SIGN), or human leukocyte antigen DR (HLA-DR). Furthermore, Gln deprivation does not affect the production of IL-12, IL-10, IL-6, or TNF.^54^ Conventional type 1 DCs (cDC1s) use the transporter protein SLC38A2 to absorb Gln in the TME, and Gln affects cDC1 antigen presentation via the folliculin (FLCN)-transcription factor EB (TFEB) axis, activating effector CD8^+^ T cell responses that are critical for tumor growth inhibition^55^ (Fig. 2). Tumor cells compete for Gln absorption with DCs by overexpressing SLC38A2, whereas SLC38A2 deficiency restores Gln levels in the TME, resulting in slower tumor growth and allowing for efficient CD8^+^ T cell recruitment and activation.^55^

As a major antigen-presenting cell in the TME, DC metabolism during activation and differentiation is poorly understood. Although Gln deprivation does not affect cytokine secretion or surface antigen expression in human monocyte-derived DCs,^54^ there is evidence that Gln is required for the expression of surface markers associated with myeloid leukocyte immune responses,^56^ implying that the role of Gln metabolism in DC differentiation may be independent of extracellular Gln supply, which requires further investigation.^54^

In summary, Gln in the TME is an important energy source for immune cell function and acts as both an anti-tumor and a pro-tumor modulator in immune modulation. Understanding the process of Gln metabolism in the TME could provide a foundation for dual-targeted tumor therapy that suppresses tumor cells while activating immune responses.

### Glutamine metabolism in other cells

In colorectal cancer TME peritoneal metastases, colorectal cancer cells outcompete adipocytes for Gln, whereas tumor cell Gln metabolism disorders induce up-regulation of Gln synthetase in cancer-associated adipocytes, and adipocyte-derived Gln promotes chemotherapy resistance by activating mTOR.^57^ Aberrant methionine metabolism in adipocytes may result in aberrant production of dimethylated histone H3 lysine 4 (H3K4me2), which may be responsible for Gln synthetase up-regulation, and lysine-specific demethylase 1 (LSD1) changes this process.^57^

Microglia are a type of glial cell that is classified as an innate immune effector cell of the central nervous system. They play an important physiological role in the normal development of the nervous system by maintaining the survival-apoptosis balance of neurons in the central nervous system and regulating neuronal homeostasis. Under pathological situations, unusually elevated GLS in microglia causes excitotoxicity via excess Glu. GLS, as a key activator of microglia, contributes to the production of proinflammatory extracellular vesicles and neuroinflammation.^58^ Furthermore, tumor cells produce glutamate, which activates AMPA-mediated microglial chemotaxis to tumor sites.^52^

Astrocytes are a type of glial cell found between nerve cell bodies and their protrusions. They support and separate nerve cells and play a role in the establishment of the blood–brain barrier. Astrocytes in normal tissues may break down glutamate, a neurotransmitter found in synapses, and are the primary pathway for neurotransmitter degradation and nervous system maintenance.^59^ Most glutamate is absorbed by astrocytes and then amidated by the ATP-dependent enzyme Gln synthetase to produce Gln. Glutamate dehydrogenase or aminotransferase converts the remaining glutamic acid to α-ketoglutarate, which is then oxidized in the TCA cycle.^59^ Research indicates that Gln synthetase suppression reduces glutamate uptake and Gln release, while TNF-α lowers Gln synthetase expression and prevents glutamate-induced Gln synthetase activation, leading to increased excitotoxicity to neurons.^60^ TGF-β also impairs Gln synthetase function in astrocytes, causing reduced Gln metabolism and enhancing N-methyl-d-aspartate receptor-mediated neurotoxicity.^61^ In addition, TNF-α activates the GLS isomer kidney type glutaminase through signal transducer and activator of transcription 3 (STAT3), resulting in elevated GLS activity.^62^ Normal astrocytes play an important role in inhibiting glioma growth and reducing neuronal damage caused by glioma glutamate release; however, as tumors grow, astrocytes' protective effects decrease.^63^

## Regulation of TME by glutamine metabolites

Immune cells are significantly impacted by the accumulation of ammonia, a by-product of urea cycle dysregulation and altered Gln metabolism within the TME.^64^^,^^65^ Glutamate, a critical nutrient for CD8^+^ T lymphocytes, undergoes mitochondrial metabolism, leading to ammonia accumulation, which promotes lysosomal alkalinization, mitochondrial hypertrophy, and eventual cellular death.^66^ Dysregulation of the sulfide transfer pathway elevates extracellular ammonia levels, inducing oxidative stress in effector T cells and leading to reduced proliferation and functional impairment.^67^^,^^68^ To sustain ammonia-neutral Gln catabolism and respond to continuous antigenic stimulation, CD8^+^ T cells rely on elevated expression of GOT1, a crucial enzyme that facilitates malic acid shuttling, thereby supporting metabolic adaptation.^69^ Thus, the effective removal of ammonia is crucial for effector T cell reactivation. Additionally, certain T cell subsets exhibit specialized metabolic mechanisms for ammonia detoxification. Long-lived memory T cells express enzymes involved in the urea and citrulline cycles, enabling efficient ammonia detoxification and facilitating memory development in response to the high-ammonia microenvironment.^70^ In macrophages, ammonia disrupts phagosome-lysosome fusion.^71^ Ammonia accumulation in the TME can lead to DC enlargement and mitochondrial impairment, directly contributing to reduced DC populations, impaired lymphocyte activation, and phagocytic dysfunction.^72^ NK cells exhibit decreased levels of mature perforin and reduced anti-tumor efficacy following ammonia exposure.^73^ In addition to ammonia accumulation inhibiting immune cell function, its metabolic byproducts, polyamines, can promote tumor cell proliferation and modulate T cell apoptosis, thereby contributing to the formation of an immunosuppressive microenvironment.^74^^,^^75^

As a key product of Gln metabolism, glutamic acid plays a crucial role in shaping the TME by linking major metabolic pathways, including the TCA cycle, fatty acid synthesis, and redox signaling. Tumor progression heavily depends on antioxidant mechanisms associated with altered metabolic pathways. The cystine/glutamate antiporter SLC7A11 (commonly known as xCT), a key mediator of the link between tumor metabolism and redox signaling, promotes glutathione synthesis by exporting intracellular glutamate in exchange for extracellular cystine.^76^ Moreover, xCT has been shown to be overexpressed in various malignancies and to play a significant role in promoting tumor growth by suppressing ferroptosis,^76^^,^^77^ metastasis,^78^ and drug resistance.^79^ Although gamma-aminobutyric acid (GABA) is widely recognized as a neurotransmitter, its role in tumor progression is increasingly acknowledged. Glutamate decarboxylase 1 (GAD1), which is overexpressed in lung adenocarcinoma, converts glutamate to GABA and regulates macrophage polarization.^80^ GABA promotes M2 macrophage polarization through activation of the STAT6 pathway and inhibits M1 polarization by suppressing the nuclear factor kappa B (NF-κB) and STAT3 pathways.^80^ Additionally, GABA induces the expression of fibroblast growth factor 2 (FGF2) in macrophages, thereby supporting tumor neovascularization.^80^ Unconventional synergistic interactions among key metabolic enzymes, including phosphomevalonate kinase (PMVK), GAD1, and acetyl-CoA acetyltransferase 1 (ACAT1), have been observed in a mouse model of hepatocellular carcinoma.^81^ Phosphorylation of PMVK enhances GAD1 activity and increases GABA synthesis.^81^ Additionally, PMVK recruits ACAT1 to convert GABA into 4-acetaminobutyric acid (4-Ac-GABA). Release of 4-Ac-GABA into the TME activates the GABAA receptor (GABAAR) on CD8^+^ T cells, thereby inhibiting protein kinase B (AKT1) signaling and suppressing CD8^+^ T cell activation and anti-tumor immune responses.^81^ Conversely, the glutamate modulator BHV-4157 significantly reduces glutamate levels in the glioma TME, decreases regulatory T cells, and promotes infiltration of non-regulatory T cells, particularly CD4^+^ T cells, thereby improving survival outcomes.^82^

## Glutamine affects the tumor mesenchyme

### Glutamine metabolism in fibroblasts

Cancer-associated fibroblasts (CAFs) are more dependent on Gln and responsive to GLS inhibition than epithelial tumor cells.^83^ CAFs absorb and use Gln from the TME in various ways. On the one hand, p62 deletion in stromal fibroblasts directly regulates activating transcription factor 4 (ATF4) stability via ubiquitin-mediated proteasomal degradation, promoting resistance to Gln deprivation.^84^ On the other hand, CAFs exhibit high levels of GLS1 and glutamate dehydrogenase-1 (GLUD1), employing Gln as the major fuel for the TCA cycle.^85^

**Figure 4:**
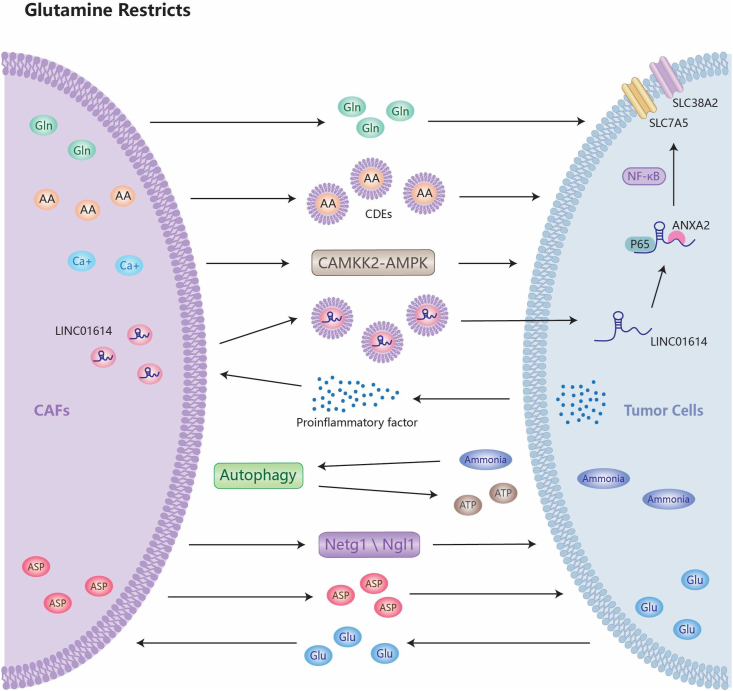
CAFs interact with tumor cells. In the absence of glutamine, CAF-derived glutamine symbiotically supports tumor cell proliferation. CAFs supply cancer cells with amino acids by secreting exosomal CDEs, which are absorbed by tumor cells, thereby completing the metabolic reprogramming process. The CAMKK2-AMPK signaling pathway boosts macropinocytic induction in CAFs when glutamine levels are low, releasing protein-derived amino acids into tumor cells with nutritional deficiencies. CAF-specific long non-coding RNA LINC01614 is released via exosomes and transferred to tumor cells, where it interacts with ANXA2 and p65 to stimulate NF-κB activation and up-regulation of glutamine transporters, SLC38A2 and SLC7A5. At the same time, tumor-derived pro-inflammatory cytokines up-regulate LINC01614 in CAFs, resulting in a feedback loop between CAFs and cancer cells. Cancer cell metabolism produces ammonia that sustains CAFs' autophagy, serving as a primary energy source to enhance mitochondrial activity in cancer cells. Through the Netg1/Ngl1 axis, CAFs supply tumor cells with critical metabolites like glutamate and glutamine. This makes it easier for cancer cells in the tumor microenvironment to get glutamine and helps tumor cells deal with nutritional stress. The transfer of glutamine-dependent aspartate and glutamate between CAFs and cancer cells is critical for the formation of aggressive tumor niches and the maintenance of pro-tumor activity. Aspartate from CAFs promotes cancer cell proliferation, while glutamate from cancer cells balances the redox status of CAFs to help the extracellular matrix change. CAF, cancer-associated fibroblast; AA, amino acid; ASP, aspartic acid; CDE, CAF-derived exosome; Glu, glutamic acid; Gln, glutamine.

CAFs provide an essential energy source for cancer cells to survive Gln deficiency in the TME, and cancer cells produce metabolites that stabilize CAFs and promote extracellular matrix remodeling. Cancer cells increase Gln production by affecting CAF metabolism with atypical nutrients of carbon and nitrogen, so CAF-derived Gln maintains cancer cell proliferation in a symbiotic manner in Gln-deficient conditions.^86^ CAFs promote cancer cell metabolic reprogramming by secreting exosomes, which are taken up by tumor cells and provide amino acids in nutrient-deficient settings. CAF-derived exosomes reduce mitochondrial oxidative phosphorylation in cancer cells, promoting glycolysis and Gln-dependent reductive carboxylation.^87^ In a Gln-depleted environment, intracellular calcium ions in CAFs increase, which activates the calcium/calmodulin-dependent protein kinase kinase 2 (CAMKK2)-AMP-activated protein kinase (AMPK) signaling pathway and amplifies macropinocytic induction in CAFs, resulting in protein-derived amino acids.^88^ These amino acids are released extracellularly to feed pancreatic ductal adenocarcinoma cells in nutrient-deficient environments.^88^ The CAF-specific long-chain non-coding RNA LINC01614, packaged by CAF-derived exosomes, directly interacts with annexin A2 (ANXA2) and p65 to promote the activation of NF-κB. This leads to the up-regulation of the Gln transporter proteins SLC38A2 and SLC7A5, which mediate Gln uptake in lung adenocarcinoma cells.^89^ Tumor-derived pro-inflammatory cytokines subsequently up-regulate LINC01614 in CAFs, forming a feed-forward loop between CAFs and cancer cells.^89^ Ammonia generated by cancer cell metabolism sustains CAF autophagy.^90^ Gln is also an important energy source for promoting mitochondrial activity in cancer cells by boosting autophagy in fibroblasts.^91^ Netrin G1-positive (NetG1^+^) CAFs assist pancreatic ductal adenocarcinoma cells in overcoming nutritional stress by feeding tumor cells with important metabolites, such as glutamate and Gln, via the NetG1/netrin-G ligand-1 (NGL1) axis, thereby enhancing the pathway for cancer cells in pancreatic ductal adenocarcinoma to get Gln from the TME and supporting pancreatic ductal adenocarcinoma survival.^92^ The Gln-dependent exchange of aspartate and glutamate between CAFs and cancer cells is crucial for shaping the pro-invasive tumor ecological niche and sustaining pro-tumorigenic activity. CAF-derived aspartate promotes cancer cell proliferation, whereas cancer-cell-derived glutamate balances CAF redox status to promote extracellular matrix remodeling^93^ (Fig. 4).

In addition to providing nutritional support to cancer cells, NetG1^+^ CAFs, a specific subpopulation of CAFs, have immunosuppressive qualities that prevent cancer cell proliferation and immune evasion. CAFs up-regulated IL15, a potent activator of NK cell activity, but nevertheless decreased NK cell-mediated death of tumor cells. This could be due to the fact that CAFs release more immunosuppressive substances, mainly TGF-β, which overwhelm NK cells' capacity to destroy tumor cells.^92^ Gln-directed migration of cancer-associated fibroblasts has been found to increase epithelial tumor invasion.^83^

### Others

Collagen and blood vessels in the tumor mesenchyme provide favorable conditions for tumor growth. Type I collagen is abundant in tumor mesenchyme and has been linked to a variety of anti- and pro-tumor effects.^94^ Tumor endothelial marker 8 (TEM8) is a cell surface protein that is overexpressed in human colorectal cancer compared with the normal endothelium.^95^ TEM8^+^ tumor mesenchymal cells may absorb type I collagen and rapidly convert it to Gln, providing an energy source for tumor cell development in nutrient-deficient situations.^96^ Vascular endothelial GLS is required for tumor angiogenesis, progression, and metastasis. Vascular endothelial GLS deficiency reduces tumor microvessel density and neovascularization, inhibiting tumor development and metastatic dissemination while increasing chemotherapy sensitivity.^97^

In summary, tumor mesenchyme serves as an energy reservoir for tumor cells by manufacturing Gln via numerous routes, resisting the TME's nutrient-deficient environment, and driving tumor proliferation and spread. Preventing tumor mesenchyme interactions with tumor cells and inhibiting its energy supply are potential options for targeted therapy.

## Targeting glutamine metabolism in the TME

### Targeting the glutamine metabolic pathway

Glutamate synthesis, absorption, and utilization in the TME are targeted to offer anti-tumor effects by directly inhibiting tumor cell metabolism, restricting stromal cell energy supply, and enhancing the TME anti-tumor immunity via many pathways.

By directly inhibiting the metabolism of tumor cells, targeting Gln metabolism slows down the growth of tumors. There have been reports of therapeutic approaches that target SLC1A5 to decrease tumor growth by depriving cancer cells of Gln, such as hepatocellular carcinoma,^98^ head and neck squamous cell carcinoma,^8^ and colon cancer.^99^ Therapeutic benefits of GLS targeting have been demonstrated in several tumor types, including endometrial cancer,^100^ clear cell ovarian carcinoma,^101^ and hepatocellular carcinoma.^102^ Furthermore, tumors, such as renal cell carcinoma,^9^ small cell lung cancer,^103^ and hepatocellular carcinoma,^104^ that overexpress MYC respond favorably to Gln metabolism inhibition. The Rb family's inhibition of Gln metabolism is involved in tumor suppressor function and could be a new target for anti-cancer therapy.^105^ Cells that have had phosphoenolpyruvate carboxykinase 2 (PCK2) knockdown become resistant to the estrogen receptor-positive (ER^+^) breast cancer development that is brought on by Gln-mediated mTORC1 pathway activation.^106^ When compared with normal pancreatic tissues or cells, pancreatic cancer showed a significant up-regulation of circ-membrane-bound O-acyltransferase domain containing 2 (circ-MBOAT2) expression. Through the miR-433-3p/GOT1 axis, circ-MBOAT2 controls carcinogenesis and Gln catabolism in pancreatic cancer, indicating that circ-MBOAT2 may be a therapeutic target for pancreatic cancer.^107^ Oral squamous cell carcinoma is inhibited by ubiquitin-specific protease 30 (USP30), which is also the target of the USP30 inhibitor MF-094; USP30 promotes Gln depletion of oral squamous cell carcinoma and inhibits apoptosis.^108^ When applied to malignant peripheral nerve sheath tumors, the new Gln antagonist prodrug Jhu395 exhibits anti-cancer activity.^109^ A potential therapeutic approach for managing Gln-dependent lung squamous cell carcinoma is to target Gln hydrolysis.^110^ DRP-104 inhibits tumor metabolism by decreasing Gln flow into the TCA cycle.^111^ Targeting highly invasive ovarian cancer cells by blocking Gln entry into the TCA cycle and less invasive ovarian cancer cells by inhibiting Gln production and STAT3 may provide novel therapeutic options for ovarian cancer.^112^

Targeting Gln metabolism disrupts communication between cancer and stromal cells, preventing energy delivery. Stromal cells require collagen to produce Gln, which provides energy for cancer cell survival. TEM8-neutralizing antibodies inhibit collagen uptake to reverse this process.^96^ CAFs create and release Gln in the TME, which is used by cancer cells as an alternative carbon and nitrogen source.^87^ Thus, inhibiting Gln uptake from CAFs by cancer cells provides a targeted method for depleting cancer cells' energy source.^113^ Several studies have found that ASCT2, a major transporter for Gln uptake by cancer cells, is vital in Gln metabolism in tumor cells. Furthermore, although it is unknown how pharmacological suppression of ASCT2 impacts lymphocyte function and consequently tumor progression, ASCT2 deletion has been observed to result in variance in distinct subsets of T cells.^28^^,^^114^ ASCT2 inhibitors have been demonstrated to limit Gln uptake and metabolism in a variety of cancers, reducing tumor growth.^115^ Tamoxifen and raloxifene also prevent the growth of ER^−^ cancers by reducing Gln uptake.^116^ CAFs and cancer cells perceive GLS1-dependent Gln catabolism and aspartate/Glu exchange via the same cotransporter (SLC1A3) as a shared metabolic program, and targeting stromal and tumor cells with GLS1 and SLC1A3 may become a viable anti-cancer therapeutic strategy.^93^ CAFs use Gln as a primary energy source rather than glucose, and inhibitors of GLS1 and GLUD1 reduce stromal cell proliferation. Thus, targeting Gln-dependent metabolic pathways may significantly impact the pro-connective tissue proliferative stroma.^85^ Although inhibiting GLS results in a Gln deprivation environment that limits tumor cell growth, this process involves an increase in glucose-dependent pyruvate carboxylase activity, making cancer cells resistant to Gln uptake and metabolic inhibition, promoting Gln-independent tumor cell growth.^117^ This process may be a crucial mechanism for addressing Gln metabolism resistance; therefore, targeting GLS and pyruvate carboxylase together may be an effective way to overcome Gln metabolism inhibition alone. Co-targeting stromal Gln synthetase and cancer cell GLS inhibits Gln-dependent cancer cell proliferation, which is controlled by the TME, and has been demonstrated to cause tumor regression in a mouse model of ovarian cancer.^86^ Ultrasound-targeted microbubble disruption works by disrupting Gln metabolic connections between CAFs and cancer cells.^118^ Targeting Gln metabolism in the host vasculature could improve clinical outcomes.^97^

Targeting Gln metabolism increases immune cell activation and stimulates anti-tumor immunity. A variety of Gln inhibitors have been demonstrated to decrease Gln uptake by tumor cells while increasing T cell killing.^119^ Blocking inositol-requiring enzyme-1alpha (IRE1α)-X-box binding protein 1 (XBP1) activation or increasing Gln transporter expression promotes mitochondrial respiration of T cells in ovarian cancer and restores anti-tumor activity.^39^ Modulating the high expression of Gln synthetase in TAMs and blocking further interaction with Tregs to counteract the immunosuppressive microenvironment could be a combined immune checkpoint therapeutic strategy for targeting Macro_APOE/CTSZ high-occupancy colon cancer or lung cancer.^45^ V-9302, a Gln transporter inhibitor, selectively limits Gln absorption by triple-negative breast cancer cells but not T cells, stimulates glutathione synthesis and Gln metabolism in T cells, and increases the activation of Th1 and CD8^+^ T cells, ultimately boosting T cell effector function.^28^ Blocking Gln metabolism in T cells gave tumor-infiltrating lymphocytes (TILs) a long-lived, memory-like state and improved their survival, proliferation, and effector functions.^31^ As a result, the combination of Gln metabolism inhibitors with anti-programmed death-ligand 1 (PD-L1) may provide improved anti-tumor efficacy.^26^^,^^31^ Gln synthetase activity is a modulator of pro-angiogenic, immunosuppressive phenotypic flipping, and pro-metastatic activities in M2-like macrophages; therefore, targeting Gln synthetase may offer an effective approach to treat cancer metastasis.^47^^,^^51^ Targeting tumor Gln metabolism reduces colony-stimulating factor 3 (CSF3), which draws myeloid-derived suppressor cells and promotes immunogenic cell death, leading to an increase in inflammatory TAMs.^120^ Furthermore, inhibiting Gln metabolism in myeloid-derived suppressor cells causes activation-induced cell death and converts myeloid-derived suppressor cells into inflammatory macrophages.^120^ CD40 activation causes Gln-to-lactate conversion in macrophages, which fine-tunes the NAD^+^/NADH ratio and boosts fatty acid oxidation (FAO)-induced anti-tumor activity.^121^ Chemotherapy-induced macrophage-derived IL-18 and l-amino acid transporter 2 (LAT2)-mediated Gln and leucine absorption increased CD47 expression by activating the mTORC1/cMyc axis, inhibiting macrophage phagocytosis of osteosarcoma cells. Targeting LAT2 increases macrophage phagocytosis and tumor cell susceptibility to chemotherapy.^122^ BVC (a photodynamic immunostimulant)-mediated potent photodynamic therapy inhibits Gln transport and glutathione synthesis by targeting ASCT2-mediated Gln metabolism, resulting in up-regulation of PDL1 and Fas, which not only increases the percentage of effector T cells and activation and immune recognition among Fas overexpressing tumor cells, but also plays an important role in the eradication of metastatic tumors by reducing immune escape of tumor cells via programmed death 1 (PD1)/PDL1 blockade.^123^ Electrodynamic treatment combined with the Gln antagonist 6-Diazo-5-oxo-l-norleucine (DON) enhances the protective immune response to electrodynamic treatment stimulation by attracting and boosting DC maturation by CD8^+^ T cells.^124^

### Targeted immunosuppression of the microenvironment

Interfering with the establishment of the immunosuppressive microenvironment and enhancing the anti-tumor activity of immune cells are potential treatment strategies. NetG1 in CAFs regulates the immunosuppressive environment in pancreatic ductal adenocarcinoma. NetG1 inhibition may alleviate the immunosuppressive phenotype via IL15, allowing NK cells to eliminate cancer cells. These data suggest that NetG1 may be a therapeutic target in pancreatic ductal adenocarcinoma.^92^ Macrophage-secreted IL-23 enhances Treg function. Blocking IL-23 may thus be a potential therapeutic strategy for clear cell renal cell carcinoma that counteracts Treg-mediated immunosuppression, induces T-cell-mediated anti-tumor immunity, and enhances the immunomodulatory effects of immune checkpoint inhibitors.^44^ LAG3, a known immunosuppressive gene, was significantly up-regulated in mRNA and protein expression levels in γδ T cells, with Gln shortage in the TME as a potential key inducer of LAG3 up-regulation. LAG3 blockade is a beneficial adjuvant combination for the adoptive metastasis of γδ T cells in hepatocellular carcinoma immunotherapy. LAG3 may be a more effective therapeutic target than PD1 and cytotoxic T lymphocyte antigen-4 (CTLA-4) in immune checkpoint therapy for hepatocellular carcinoma patients. However, further mouse and clinical trials are required to support this viewpoint.^43^

As a key energy substance for T cells, Gln plays an unquestionable role in the immune function of T cells. However, ammonia, as an important product of Gln metabolism, has been reported to be a key factor in effector CD8^+^ T cell death.^125^ With the high energy metabolism of active CD8^+^ T cells, intracellular ammonia levels gradually increase, eventually leading to cell death.^125^ This groundbreaking discovery provides a new idea for enhancing effector T cell survival, reducing effector T cell death caused by ammonia toxicity. The causes of intracellular ammonia accumulation, how ammonia accumulation precisely regulates the mechanism of T cell death, and whether ammonia-induced cell death exists in other stromal cells may be important directions for future work.^125^

In conclusion, targeting Gln balances the power of cancer cells and tumor stromal components in the TME from metabolic and immunological perspectives, thereby changing the metabolic tendency in the TME and ultimately impairing tumor growth. It is accomplished by directly impeding tumor cell metabolism, blocking energy supply from the tumor mesenchyme, and fostering the TME's anti-tumor immune mechanism.

### Clinical trials and safety

Targeting Gln metabolism has shown great potential for the treatment of solid tumors. Currently, several Gln metabolism-related targeted agents have entered clinical studies, including GLS inhibitors, Gln antagonists, CD40 agonists, and LAG3 blockers (Table 1). However, given that Gln plays a crucial role in the normal function of multiple organs, such as the liver, brain, and intestines,^126^^,^^127^ the safety of targeted Gln therapy warrants careful consideration.

**Table 1:** Summary of clinical trials of glutamine metabolism-related targets in solid tumors.

Targets	Glutamine-targeting agents	Mechanisms	Tumor type	Clinical trial
Glutaminase	Telaglenastat (CB-839)	Inhibits glutaminase	Advanced/metastatic solid tumors, fumarate hydratase-deficient tumors, succinate dehydrogenase-deficient gastrointestinal stromal tumors, IDH1 and IDH2 mutations, amplifications in the cMyc gene, KRAS gene mutation, KEAP1 gene mutation, NRF2 gene mutation, NFE2L2 gene mutation	NCT05521997, NCT02071862, NCT03872427, NCT03875313, NCT03965845, NCT03057600, NCT02771626, NCT03163667, NCT03428217, NCT04265534
Glutamine antagonists	DRP-104	Reduces glutamine uptake and breakdown	Advanced/metastatic solid tumors	NCT06027086, NCT04471415
CD40	APX005M (sotigalimab)	CD40 activation causes macrophages to convert glutamine, which balances the NAD^+^/NADH ratio and increases FAO-induced anti-tumor activity	Advanced/metastatic solid tumors	NCT02482168, NCT03123783, NCT04337931, NCT03597282
	JNJ-64457107		Advanced solid tumors	NCT02829099
	RO7300490		Solid tumors	NCT04857138
	Selicrelumab (RO7009789)		Advanced/metastatic solid tumors	NCT02304393, NCT02665416
	ADC-1013		Solid tumors	NCT02379741
	BMS-986484		Advanced solid tumors	NCT06544655
	CP-870,893		Solid tumors	NCT00711191
	SEA-CD40		Advanced/metastatic solid tumors, lymphoma	NCT04993677, NCT02376699
	CDX-1140		Advanced/metastatic solid tumors, lymphoma	NCT03329950
LAG3	Sym022	LAG3 blockage responds to γδT cell-mediated immunosuppression in the tumor microenvironment of glutamine deficiency	Advanced/metastatic solid tumors, lymphoma	NCT03489369, NCT03311412, NCT04641871
	INCA32459-101		Advanced malignancies	NCT05577182
	REGN3767		Malignancies	NCT03005782
	Relatlimab (BMS-986016)		Advanced/metastatic solid tumors, lymphoma	NCT02966548, NCT01968109, NCT02658981, NCT06400264, NCT05498480, NCT05134948, NCT04080804, NCT03743766, NCT03459222, NCT03623854, NCT03607890, NCT03662659, NCT02061761, NCT05034536, NCT05148546, NCT04658147, NCT03026140, NCT04326257, NCT03642067, NCT06325683
	AK129		Advanced malignant tumors, solid tumors	NCT05645276, NCT06586294
	EMB-02		Advanced solid tumors	NCT04618393
	HLX26		Adult solid tumors, lymphoma	NCT05078593, NCT05400265, NCT05787613, NCT05584137
	Tuparstobar (INCAGN02385)		Solid tumors, lymphoma	NCT04370704, NCT05287113, NCT04586244, NCT03538028, NCT06056895
	ABL501		Advanced solid tumors	NCT05101109
	FS118		Advanced/metastatic solid tumors	NCT03440437
	Tobemstomig (RO7247669)		Advanced/metastatic solid tumors	NCT04140500, NCT04785820, NCT05645692
	Eftilagimod alpha (IMP321)		Advanced/metastatic solid tumors	NCT04811027, NCT03625323, NCT06128863, NCT05747794, NCT00732082, NCT00349934, NCT04252768, NCT00351949
	Fianlimab (REGN3767)		Solid tumors	NCT05352672, NCT05800015, NCT06246916, NCT05785767, NCT05608291, NCT06571708, NCT06205836
	XmAb22841		Advanced/metastatic solid tumors	NCT03849469, NCT05695898
	TSR-033		Neoplasms	NCT03250832
	Tebotelimab (MGD013)		Advanced solid tumors, hematologic neoplasms	NCT03219268, NCT04129320
	Favezelimab		Solid tumors	NCT06036836
	IBI110		Solid tumors	NCT06494943

Targeted GLS inhibition can directly suppress Gln catabolism, preventing Gln from supplying intermediate metabolites and nutrients to the TME metabolic network. Phase I clinical trials have shown that a biologically active dose of the GLS inhibitor CB-839 in combination with the chemotherapy drug capecitabine is well tolerated.^128^ The Gln antagonist DON significantly impairs cellular Gln metabolism; however, its clinical application is limited by dose-dependent gastrointestinal toxicity. To address this limitation, the DON precursor DRP-104 has been developed.^111^ By encouraging anti-tumor CD8^+^ and CD4^+^ T cell responses, DRP-104 can inhibit Gln-dependent nucleotide synthesis and suppress the growth of Kelch-like ECH-associated protein 1 (KEAP1)-mutant tumors.^129^ The dual effects of DRP-104 in blocking metabolism and enhancing T cell function have been demonstrated in animal experiments,^31^ warranting further investigation in clinical trials. In addition, DON-derived Gln antagonists, such as JHU395 and JHU083, have demonstrated favorable tolerability in preclinical studies.^109^^,^^120^ CD40 agonists modulate immune cells within various components of the TME, including macrophages, T cells, and DCs, and have been shown to be safe and effective in multiple clinical studies.^130^, ^131^, ^132^ LAG3 blockers counteract immunosuppression within the TME and exert potent anti-tumor effects.^43^ The combination of LAG3 blockade and PD-L1 inhibition has demonstrated a stronger immune response than PD-L1 inhibition alone, with an encouraging safety profile.^133^

## Future perspective

### Potential biomarkers of patient stratification in glutamine therapy

Due to the heterogeneity in Gln metabolism mechanisms among patients with tumors, stratification is crucial, as personalized treatment tailored to tumor histological subtype and specific driver gene alterations may optimize therapeutic outcomes. The glutamate-to-Gln ratio indicates the extent of GLS-driven catabolic activity. A significant increase was observed in 88% of ER^+^ tumors and 56% of ER^−^ tumors relative to normal breast tissue, indicating the substantial effectiveness of GLS inhibitors in these cells.^134^ Breast cancer gene (BRCA)-mutated cancers generally exhibit proliferation and invasion dependent on Gln metabolism and may serve as potential biomarkers for response to GLS inhibitor therapy.^135^ Despite the efficacy of epidermal growth factor receptor-tyrosine kinase inhibitors (EGFR-TKIs) in treating patients with EGFR-mutant lung cancer, the issue of drug resistance stemming from Gln metabolism, particularly related to the EGFR T790M mutation and p53 mutation, persists as a major challenge. The combination of the EGFR-targeting agent gefitinib and the SLC7A5 inhibitor BCH has been shown to effectively overcome this resistance challenge.^136^ Moreover, KRAS-mutant lung cancer exhibits sensitivity to GLS inhibitors, and the deletion of neurofibromatosis type-1 (Nf1) can increase Kras-mutant susceptibility to GLS inhibition.^137^ Mutations in KEAP1 and abnormal expression of BRCA1-associated protein-1 (BAP1) facilitate the identification of cancers with elevated levels of the Gln transporter SLC7A11, potentially benefiting patients through chemoradiotherapy combined with SLC7A11 inhibition.^136^ Keap1 mutations initiate the nuclear factor erythroid 2-related factor 2 (Nrf2) antioxidant program, facilitate Kras-driven lung cancer advancement, and lead to a dependency on Gln catabolism.^138^^,^^139^ KEAP1/NRF2 mutation-induced cellular redox imbalances have been identified as potential markers for therapy involving combination with GLS inhibitors.^140^ MYCN amplification-induced cellular autonomous damage and ATF4-mediated microenvironmental stress synergistically regulate ASCT2 activation to sustain sufficient Gln levels essential for TCA cycle recovery.^141^^,^^142^ Thus, ATF4 and ASCT2 are recognized as new biomarkers for patient stratification and prognostic prediction. However, the potential of patient stratification in Gln metabolism therapy requires further investigation. Although potential biomarkers for patient stratification have been increasingly identified, further direct *in vitro* and *in vivo* validation is warranted, given the dynamic epigenetic regulation of gene expression.

Currently, enzymatic detection technologies, such as immunohistochemistry, enzyme-linked immunosorbent assay, high-performance liquid chromatography, and other technologies for determining Gln content, are widely utilized for the *in vitro* detection of Gln metabolism levels. Genetic testing of cellular tumor tissues and modeling of the TME using organoids are crucial for the personalized treatment of clinical solid tumors.^143^^,^^144^ *In vitro* experiments serve as a critical method for validating novel therapeutics targeting Gln metabolism, although they remain limited in their ability to visualize the heterogeneity and dynamics of the local tumor environment.^145^ Therefore, *in vivo* monitoring of Gln metabolism is essential for a comprehensive understanding of its potential as a biomarker. Metabolic imaging techniques, such as 1H-MRS, hyperpolarized 13C-MRS, deuterium metabolic imaging, mass spectrometry imaging, and fluorescence imaging, have been reported as effective tools for assessing Gln metabolism in glioma *in vivo*,^145^^,^^146^ and are anticipated to become important tools in cancer research and clinical treatment.

### Resistance to glutamine-targeted therapies

While Gln-targeted therapy has proven effective in enhancing various tumor treatments, resistance to Gln-targeted therapy has been documented,^117^^,^^147^ highlighting the need for strategies to overcome this challenge. We outline the mechanisms underlying resistance to Gln-targeted treatments and propose potential research directions to address this issue.

To counteract the suppression of Gln metabolism, tumors utilize amino acid transporters and activate alternative metabolic pathways to maintain essential resources for growth, leading to metabolic adaptability.^148^ Research indicates that pyruvate carboxylase-mediated glucose-dependent supplementation can enhance oxaloacetate levels, allowing cells to attain Gln independence.^117^ The up-regulation of alternative metabolic pathways in the TCA cycle, including GLS2 and amidotransferase, may partially offset the reduction of GLS activity in Gln metabolism, representing a potential mechanism of treatment resistance to Gln degradation.^149^ Endogenously synthesized pyruvate released by triple-negative breast cancer cell lines acts in a paracrine manner, leading to the resistance of recipient cells to GLS inhibition.^150^ Elevated expression of critical genes associated with mitochondrial oxidation (CPT1B, CPT2, and CRAT) and activation of the FAO pathway has been observed in drug-resistant cell lines, namely in CB-839-resistant breast cancer and pancreatic ductal adenocarcinoma.^147^^,^^151^ Consequently, the concurrent inhibition of Gln-targeted therapy alongside other metabolic pathways represents a key approach to overcoming drug resistance. The simultaneous inhibition of GLS and carnitine palmitoyltransferase has demonstrated efficacy in reducing resistance to GLS inhibition in triple-negative breast cancer cells.^147^ Animal studies have demonstrated that the suppression of GLS and hexokinase 2 (Hk2), which disrupts both Gln and glucose metabolism, can significantly impede tumor progression.^149^ Nonetheless, despite the promising outcomes of combination inhibition in cellular investigations and animal models, robust clinical trial evidence remains limited, necessitating further investigation.

The emergence and progression of cancers are influenced not only by Gln metabolism but also by the contributions of other metabolic pathways within the TME. The energy deficit and hypoxic conditions in the TME result in lactate release via the glycolytic pathway, which promotes endothelial cell proliferation and enhances tumor vascularization.^152^ Lactate can elevate the expression of PD-L1 through the activation of NF-κB, thereby facilitating M2-type polarization of macrophages and inducing immunosuppressive effects.^153^ The integration of Gln-targeted treatment with angiogenesis inhibition and immunosuppression may yield superior outcomes. The dual targeting of Gln metabolism and PD-L1 has demonstrated efficacy in suppressing tumor progression in murine models.^154^ The significant importance of Gln-targeted therapy and the dual approach involving essential components of the TME in cancer treatment warrants further investigation.

## Conclusion

The TME's interaction with tumors is linked to Gln metabolism. This research demonstrates the therapeutic importance of targeting the Gln metabolism pathway by investigating the functional changes in tumor stromal components within the TME induced by Gln metabolism, including stromal cells and tumor mesenchymal components. Moreover, further research is needed into the mechanisms of Gln metabolism, metabolic complexity, and metabolic therapy to fully realize the clinical potential of Gln metabolism therapy.

## CRediT authorship contribution statement

**Zizhuo Li:** Writing – review & editing, Writing – original draft, Visualization. **Jiapeng Deng:** Writing – review & editing. **Hai Wang:** Writing – review & editing. **Tao Liu:** Writing – review & editing. **Yuyang Zhou:** Writing – review & editing. **Pei Ouyang:** Writing – review & editing. **Xuan Liang:** Writing – review & editing. **Xian Zhang:** Writing – review & editing, Supervision. **Songtao Qi:** Supervision, Writing – review & editing. **Yaomin Li:** Supervision, Writing – review & editing, Funding acquisition.

## Funding

This study was supported by the 10.13039/501100001809National Natural Science Foundation of China (No. 82203368), Science and Technology Program of Guangzhou, Guangdong, China (No. 2025A04J5139), President Foundation of Nanfang Hospital, Southern Medical University, Guangdong, China (No. 2023H020), 10.13039/501100003453Natural Science Foundation of Guangdong Province, China (No. 2023A1515011775), and College Students' Innovative Entrepreneurial Training Plan Program (China) (No. S202312121119).

## Conflict of interests

The authors have declared that no competing interest exists.

## References

[1] Wang B., Pei J., Xu S., Liu J., Yu J. (2024). A glutamine tug-of-war between cancer and immune cells: recent advances in unraveling the ongoing battle. J Exp Clin Cancer Res.

[2] Faiena I., Ueno D., Shuch B. (2019). Glutamine and the tumor immune microenvironment. Eur Urol.

[3] Fernandez-Marcos P.J., Serrano M. (2013). Sirt4: the glutamine gatekeeper. Cancer Cell.

[4] Keulers T.G., Koch A., van Gisbergen M.W. (2022). ATG12 deficiency results in intracellular glutamine depletion, abrogation of tumor hypoxia and a favorable prognosis in cancer. Autophagy.

[5] Li X., Peng X., Li Y. (2024). Glutamine addiction in tumor cell: oncogene regulation and clinical treatment. Cell Commun Signal.

[6] Fahr M.J., Kornbluth J., Blossom S., Schaeffer R., Klimberg V.S., Vars Research Award Harry M. (1994). Glutamine enhances immunoregulation of tumor growth. JPEN J Parenter Enteral Nutr.

[7] Li L.B., Fang T.Y., Xu W.J. (2021). Oral glutamine inhibits tumor growth of gastric cancer bearing mice by improving immune function and activating apoptosis pathway. Tissue Cell.

[8] Zhang Z., Liu R., Shuai Y. (2020). ASCT2 (SLC1A5)-dependent glutamine uptake is involved in the progression of head and neck squamous cell carcinoma. Br J Cancer.

[9] Shroff E.H., Eberlin L.S., Dang V.M. (2015). MYC oncogene overexpression drives renal cell carcinoma in a mouse model through glutamine metabolism. Proc Natl Acad Sci U S A.

[10] Suzuki S., Tanaka T., Poyurovsky M.V. (2010). Phosphate-activated glutaminase (GLS2), a p53-inducible regulator of glutamine metabolism and reactive oxygen species. Proc Natl Acad Sci U S A.

[11] Suzuki S., Venkatesh D., Kanda H. (2022). GLS2 is a tumor suppressor and a regulator of ferroptosis in hepatocellular carcinoma. Cancer Res.

[12] Hensley C.T., Wasti A.T., DeBerardinis R.J. (2013). Glutamine and cancer: cell biology, physiology, and clinical opportunities. J Clin Investig.

[13] Chapman V.M., Ruddle F.H. (1972). Glutamate oxaloacetate transaminase (got) genetics in the mouse: polymorphism of got-1. Genetics.

[14] Meléndez-Rodríguez F., Urrutia A.A., Lorendeau D. (2019). HIF1α suppresses tumor cell proliferation through inhibition of aspartate biosynthesis. Cell Rep.

[15] Fasoulakis Z., Koutras A., Ntounis T. (2023). Ovarian cancer and glutamine metabolism. Int J Mol Sci.

[16] Wei Y., Tang X., Ren Y. (2021). An RNA-RNA crosstalk network involving HMGB1 and RICTOR facilitates hepatocellular carcinoma tumorigenesis by promoting glutamine metabolism and impedes immunotherapy by PD-L1^+^ exosomes activity. Signal Transduct Target Ther.

[17] Tardito S., Oudin A., Ahmed S.U. (2015). Glutamine synthetase activity fuels nucleotide biosynthesis and supports growth of glutamine-restricted glioblastoma. Nat Cell Biol.

[18] Hoerner C.R., Chen V.J., Fan A.C. (2019). The ‘Achilles heel’ of metabolism in renal cell carcinoma: glutaminase inhibition as a rational treatment strategy. Kidney Cancer.

[19] Yang C., Ko B., Hensley C.T. (2014). Glutamine oxidation maintains the TCA cycle and cell survival during impaired mitochondrial pyruvate transport. Mol Cell.

[20] Estévez-García I.O., Cordoba-Gonzalez V., Lara-Padilla E. (2014). Glucose and glutamine metabolism control by APC and SCF during the G1-to-S phase transition of the cell cycle. J Physiol Biochem.

[21] Filipp F.V., Ratnikov B., De Ingeniis J., Smith J.W., Osterman A.L., Scott D.A. (2012). Glutamine-fueled mitochondrial metabolism is decoupled from glycolysis in melanoma. Pigment Cell Melanoma Res.

[22] Leone R.D., Powell J.D. (2020). Metabolism of immune cells in cancer. Nat Rev Cancer.

[23] Wik J.A., Chowdhury A., Kolan S. (2022). Endogenous glutamine is rate-limiting for anti-CD3 and anti-CD28 induced CD4^+^ T-cell proliferation and glycolytic activity under hypoxia and normoxia. Biochem J.

[24] Carr E.L., Kelman A., Wu G.S. (2010). Glutamine uptake and metabolism are coordinately regulated by ERK/MAPK during T lymphocyte activation. J Immunol.

[25] Wang R., Dillon C.P., Shi L.Z. (2011). The transcription factor Myc controls metabolic reprogramming upon T lymphocyte activation. Immunity.

[26] Huang R., Wang H., Hong J. (2023). Targeting glutamine metabolic reprogramming of SLC7A5 enhances the efficacy of anti-PD-1 in triple-negative breast cancer. Front Immunol.

[27] Swamy M., Pathak S., Grzes K.M. (2016). Glucose and glutamine fuel protein O-GlcNAcylation to control T cell self-renewal and malignancy. Nat Immunol.

[28] Edwards D.N., Ngwa V.M., Raybuck A.L. (2021). Selective glutamine metabolism inhibition in tumor cells improves antitumor T lymphocyte activity in triple-negative breast cancer. J Clin Investig.

[29] Suzuki J., Yamada T., Inoue K. (2018). The tumor suppressor menin prevents effector CD8 T-cell dysfunction by targeting mTORC1-dependent metabolic activation. Nat Commun.

[30] Chen J., Wang R., Liu Z. (2022). Unbalanced glutamine partitioning between CD8T cells and cancer cells accompanied by immune cell dysfunction in hepatocellular carcinoma. Cells.

[31] Leone R.D., Zhao L., Englert J.M. (2019). Glutamine blockade induces divergent metabolic programs to overcome tumor immune evasion. Science.

[32] Villanueva M.T. (2020). Cancer cells' loss is T cells' gain. Nat Rev Drug Discov.

[33] Nabe S., Yamada T., Suzuki J. (2018). Reinforce the antitumor activity of CD8^+^ T cells via glutamine restriction. Cancer Sci.

[34] Abu Eid R., Ahmad S., Lin Y. (2017). Enhanced therapeutic efficacy and memory of tumor-specific CD8 T cells by *ex vivo* PI3K-δ inhibition. Cancer Res.

[35] Abu Eid R., Friedman K.M., Mkrtichyan M. (2015). Akt1 and-2 inhibition diminishes terminal differentiation and enhances central memory CD8^+^ T-cell proliferation and survival. Oncoimmunology.

[36] Kim E.H., Sullivan J.A., Plisch E.H. (2012). Signal integration by Akt regulates CD8 T cell effector and memory differentiation. J Immunol.

[37] Li Q., Rao R., Vazzana J. (2012). Regulating mammalian target of rapamycin to tune vaccination-induced CD8^+^ T cell responses for tumor immunity. J Immunol.

[38] Cao S., Hung Y.W., Wang Y.C. (2022). Glutamine is essential for overcoming the immunosuppressive microenvironment in malignant salivary gland tumors. Theranostics.

[39] Song M., Sandoval T.A., Chae C.S. (2018). IRE1α-XBP1 controls T cell function in ovarian cancer by regulating mitochondrial activity. Nature.

[40] Matheson L.S., Petkau G., Sáenz-Narciso B. (2022). Multiomics analysis couples mRNA turnover and translational control of glutamine metabolism to the differentiation of the activated CD4^+^ T cell. Sci Rep.

[41] Ron-Harel N., Ghergurovich J.M., Notarangelo G. (2019). T cell activation depends on extracellular alanine. Cell Rep.

[42] Bradley C.A. (2018). IL-23 links glutamine addiction and immune function. Nat Rev Urol.

[43] He W., Hu Y., Chen D. (2022). Hepatocellular carcinoma-infiltrating γδ T cells are functionally defected and allogenic Vδ2^+^ γδ T cell can be a promising complement. Clin Transl Med.

[44] Fu Q., Xu L., Wang Y. (2019). Tumor-associated macrophage-derived interleukin-23 interlinks kidney cancer glutamine addiction with immune evasion. Eur Urol.

[45] Wei J., Yu W., Chen J. (2023). Single-cell and spatial analyses reveal the association between gene expression of glutamine synthetase with the immunosuppressive phenotype of APOE+CTSZ+TAM in cancers. Mol Oncol.

[46] Jiang W., Jiang Y., Zhang X., Mu H., Song Y., Zhao H. (2023). Metabolomic analysis reveals the influence of HMBOX1 on RAW_264.7_ cells proliferation based on UPLC-MS/MS. BMC Genom.

[47] Menga A., Favia M., Spera I. (2021). N-acetylaspartate release by glutaminolytic ovarian cancer cells sustains protumoral macrophages. EMBO Rep.

[48] Wang Y., Wang D., Yang L., Zhang Y. (2022). Metabolic reprogramming in the immunosuppression of tumor-associated macrophages. Chin Med J (Engl).

[49] Hu X., Ma Z., Xu B. (2023). Glutamine metabolic microenvironment drives M2 macrophage polarization to mediate trastuzumab resistance in HER2-positive gastric cancer. Cancer Commun.

[50] Liu P.S., Wang H., Li X. (2017). α-ketoglutarate orchestrates macrophage activation through metabolic and epigenetic reprogramming. Nat Immunol.

[51] Palmieri E.M., Menga A., Martín-Pérez R. (2017). Pharmacologic or genetic targeting of glutamine synthetase skews macrophages toward an M1-like phenotype and inhibits tumor metastasis. Cell Rep.

[52] Choi J., Stradmann-Bellinghausen B., Yakubov E., Savaskan N.E., Régnier-Vigouroux A. (2015). Glioblastoma cells induce differential glutamatergic gene expressions in human tumor-associated microglia/macrophages and monocyte-derived macrophages. Cancer Biol Ther.

[53] Møller S.H., Wang L., Ho P.C. (2022). Metabolic programming in dendritic cells tailors immune responses and homeostasis. Cell Mol Immunol.

[54] Schoeppe R., Babl N., Decking S.M. (2023). Glutamine synthetase expression rescues human dendritic cell survival in a glutamine-deprived environment. Front Oncol.

[55] Guo C., You Z., Shi H. (2023). SLC38A2 and glutamine signalling in cDC1s dictate anti-tumour immunity. Nature.

[56] Spittler A., Winkler S., Götzinger P. (1995). Influence of glutamine on the phenotype and function of human monocytes. Blood.

[57] Zhang X., Li Q., Du A. (2021). Adipocytic glutamine synthetase upregulation via altered histone methylation promotes 5FU chemoresistance in peritoneal carcinomatosis of colorectal cancer. Front Oncol.

[58] Ding L., Xu X., Li C., Wang Y., Xia X., Zheng J.C. (2021). Glutaminase in microglia: a novel regulator of neuroinflammation. Brain Behav Immun.

[59] de Ruiter Swain J., Michalopoulou E., Noch E.K., Lukey M.J., Van Aelst L. (2023). Metabolic partitioning in the brain and its hijacking by glioblastoma. Genes Dev.

[60] Zou J., Wang Y.X., Dou F.F. (2010). Glutamine synthetase down-regulation reduces astrocyte protection against glutamate excitotoxicity to neurons. Neurochem Int.

[61] Chao C.C., Hu S., Tsang M. (1992). Effects of transforming growth factor-beta on murine astrocyte glutamine synthetase activity. Implications in neuronal injury. J Clin Investig.

[62] Milewski K., Bogacińska-Karaś M., Hilgier W., Albrecht J., Zielińska M. (2019). TNFα increases STAT3-mediated expression of glutaminase isoform KGA in cultured rat astrocytes. Cytokine.

[63] Yao P.S., Kang D.Z., Lin R.Y., Ye B., Wang W., Ye Z.C. (2014). Glutamate/glutamine metabolism coupling between astrocytes and glioma cells: neuroprotection and inhibition of glioma growth. Biochem Biophys Res Commun.

[64] Lee J.S., Adler L., Karathia H. (2018). Urea cycle dysregulation generates clinically relevant genomic and biochemical signatures. Cell.

[65] Spinelli J.B., Yoon H., Ringel A.E., Jeanfavre S., Clish C.B., Haigis M.C. (2017). Metabolic recycling of ammonia via glutamate dehydrogenase supports breast cancer biomass. Science.

[66] Zhang H., Liu J., Yuan W. (2024). Ammonia-induced lysosomal and mitochondrial damage causes cell death of effector CD8^+^ T cells. Nat Cell Biol.

[67] Bell H.N., Huber A.K., Singhal R. (2023). Microenvironmental ammonia enhances T cell exhaustion in colorectal cancer. Cell Metab.

[68] Bian Y., Li W., Kremer D.M. (2020). Cancer SLC43A2 alters T cell methionine metabolism and histone methylation. Nature.

[69] Weisshaar N., Ma S., Ming Y. (2023). The malate shuttle detoxifies ammonia in exhausted T cells by producing 2-ketoglutarate. Nat Immunol.

[70] Tang K., Zhang H., Deng J. (2023). Ammonia detoxification promotes CD8^+^ T cell memory development by urea and citrulline cycles. Nat Immunol.

[71] Gordon A.H., Hart P.D., Young M.R. (1980). Ammonia inhibits phagosome-lysosome fusion in macrophages. Nature.

[72] Luo C., Shen G., Liu N. (2014). Ammonia drives dendritic cells into dysfunction. J Immunol.

[73] Domagala J., Grzywa T.M., Baranowska I. (2025). Ammonia suppresses the antitumor activity of natural killer cells and T cells by decreasing mature perforin. Cancer Res.

[74] Holbert C.E., Cullen M.T., Casero RA Jr, Stewart T.M. (2022). Polyamines in cancer: integrating organismal metabolism and antitumour immunity. Nat Rev Cancer.

[75] Ye Q., Li D., Zou Y., Yuan Y. (2025). The role and treatment strategies of ammonia-related metabolism in tumor microenvironment. Curr Gene Ther.

[76] Arensman M.D., Yang X.S., Leahy D.M. (2019). Cystine-glutamate antiporter xCT deficiency suppresses tumor growth while preserving antitumor immunity. Proc Natl Acad Sci U S A.

[77] Koppula P., Zhuang L., Gan B. (2021). Cystine transporter SLC7A11/xCT in cancer: ferroptosis, nutrient dependency, and cancer therapy. Protein Cell.

[78] Ruiu R., Cossu C., Iacoviello A. (2023). Cystine/glutamate antiporter xCT deficiency reduces metastasis without impairing immune system function in breast cancer mouse models. J Exp Clin Cancer Res.

[79] Miyoshi S., Tsugawa H., Matsuzaki J. (2018). Inhibiting xCT improves 5-fluorouracil resistance of gastric cancer induced by CD44 variant 9 expression. Anticancer Res.

[80] Dong Y., Wang G., Nie D. (2024). Tumor-derived GABA promotes lung cancer progression by influencing TAMs polarization and neovascularization. Int Immunopharmacol.

[81] Zhou X., Chen Z., Yu Y. (2024). Increases in 4-acetaminobutyric acid generated by phosphomevalonate kinase suppress CD8^+^ T cell activation and allow tumor immune escape. Adv Sci (Weinh).

[82] Medikonda R., Choi J., Pant A. (2021). Synergy between glutamate modulation and anti-programmed cell death protein 1 immunotherapy for glioblastoma. J Neurosurg.

[83] Mestre-Farrera A., Bruch-Oms M., Peña R. (2021). Glutamine-directed migration of cancer-activated fibroblasts facilitates epithelial tumor invasion. Cancer Res.

[84] Linares J.F., Cordes T., Duran A. (2017). ATF4-induced metabolic reprograming is a synthetic vulnerability of the p62-deficient tumor stroma. Cell Metab.

[85] Knudsen E.S., Balaji U., Freinkman E., McCue P., Witkiewicz A.K. (2016). Unique metabolic features of pancreatic cancer stroma: relevance to the tumor compartment, prognosis, and invasive potential. Oncotarget.

[86] Yang L., Achreja A., Yeung T.L. (2016). Targeting stromal glutamine synthetase in tumors disrupts tumor microenvironment-regulated cancer cell growth. Cell Metab.

[87] Zhao H., Yang L., Baddour J. (2016). Tumor microenvironment derived exosomes pleiotropically modulate cancer cell metabolism. eLife.

[88] Zhang Y., Recouvreux M.V., Jung M. (2021). Macropinocytosis in cancer-associated fibroblasts is dependent on CaMKK2/ARHGEF2 signaling and functions to support tumor and stromal cell fitness. Cancer Discov.

[89] Liu T., Han C., Fang P. (2022). Cancer-associated fibroblast-specific lncRNA LINC01614 enhances glutamine uptake in lung adenocarcinoma. J Hematol Oncol.

[90] Sotgia F., Martinez-Outschoorn U.E., Pavlides S., Howell A., Pestell R.G., Lisanti M.P. (2011). Understanding the Warburg effect and the prognostic value of stromal caveolin-1 as a marker of a lethal tumor microenvironment. Breast Cancer Res.

[91] Ko Y.H., Lin Z., Flomenberg N. (2011). Glutamine fuels a vicious cycle of autophagy in the tumor stroma and oxidative mitochondrial metabolism in epithelial cancer cells: implications for preventing chemotherapy resistance. Cancer Biol Ther.

[92] Francescone R., Vendramini-Costa D.B., Franco-Barraza J. (2021). Netrin G1 promotes pancreatic tumorigenesis through cancer-associated fibroblast-driven nutritional support and immunosuppression. Cancer Discov.

[93] Bertero T., Oldham W.M., Grasset E.M. (2019). Tumor-stroma mechanics coordinate amino acid availability to sustain tumor growth and malignancy. Cell Metab.

[94] Proia D.A., Kuperwasser C. (2005). Stroma: tumor agonist or antagonist. Cell Cycle.

[95] St Croix B., Rago C., Velculescu V. (2000). Genes expressed in human tumor endothelium. Science.

[96] Hsu K.S., Dunleavey J.M., Szot C. (2022). Cancer cell survival depends on collagen uptake into tumor-associated stroma. Nat Commun.

[97] Ngwa V.M., Edwards D.N., Hwang Y. (2022). Loss of vascular endothelial glutaminase inhibits tumor growth and metastasis, and increases sensitivity to chemotherapy. Cancer Res Commun.

[98] Jiang J., Dong W., Zhang W. (2023). LncRNA *SLC1A5-AS/MZF1/ASCT2* axis contributes to malignant progression of hepatocellular carcinoma. Discov Med.

[99] Hara Y., Minami Y., Yoshimoto S. (2020). Anti-tumor effects of an antagonistic mAb against the ASCT2 amino acid transporter on KRAS-mutated human colorectal cancer cells. Cancer Med.

[100] Guo H., Li W., Pan G. (2023). The glutaminase inhibitor compound 968 exhibits potent *in vitro* and *in vivo* anti-tumor effects in endometrial cancer. Anti Cancer Agents Med Chem.

[101] Wu S., Fukumoto T., Lin J. (2021). Targeting glutamine dependence through GLS1 inhibition suppresses ARID1A-inactivated clear cell ovarian carcinoma. Nat Cancer.

[102] Li Y., Li B., Xu Y. (2022). GOT2 silencing promotes reprogramming of glutamine metabolism and sensitizes hepatocellular carcinoma to glutaminase inhibitors. Cancer Res.

[103] Munksgaard Thorén M., Vaapil M., Staaf J. (2017). Myc-induced glutaminolysis bypasses HIF-driven glycolysis in hypoxic small cell lung carcinoma cells. Oncotarget.

[104] Xu K., Ding J., Zhou L. (2022). SMYD2 promotes hepatocellular carcinoma progression by reprogramming glutamine metabolism via c-Myc/GLS1 axis. Cells.

[105] Reynolds M.R., Lane A.N., Robertson B. (2014). Control of glutamine metabolism by the tumor suppressor Rb. Oncogene.

[106] Hsu H.P., Chu P.Y., Chang T.M. (2023). Mitochondrial phosphoenolpyruvate carboxykinase promotes tumor growth in estrogen receptor-positive breast cancer via regulation of the mTOR pathway. Cancer Med.

[107] Zhou X., Liu K., Cui J. (2021). Circ-MBOAT2 knockdown represses tumor progression and glutamine catabolism by miR-433-3p/GOT1 axis in pancreatic cancer. J Exp Clin Cancer Res.

[108] Zhang X., Han Y., Liu S. (2022). MF-094 nanodelivery inhibits oral squamous cell carcinoma by targeting USP30. Cell Mol Biol Lett.

[109] Lemberg K.M., Zhao L., Wu Y. (2020). The novel glutamine antagonist prodrug JHU395 has antitumor activity in malignant peripheral nerve sheath tumor. Mol Cancer Therapeut.

[110] Ye X., Zhou Q., Matsumoto Y. (2016). Inhibition of glutaminolysis inhibits cell growth via down-regulating Mtorc1 signaling in lung squamous cell carcinoma. Anticancer Res.

[111] Rais R., Lemberg K.M., Tenora L. (2022). Discovery of DRP-104, a tumor-targeted metabolic inhibitor prodrug. Sci Adv.

[112] Yang L., Moss T., Mangala L.S. (2014). Metabolic shifts toward glutamine regulate tumor growth, invasion and bioenergetics in ovarian cancer. Mol Syst Biol.

[113] Jung J.G., Le A. (2021). Targeting metabolic cross talk between cancer cells and cancer-associated fibroblasts. Adv Exp Med Biol.

[114] Nakaya M., Xiao Y., Zhou X. (2014). Inflammatory T cell responses rely on amino acid transporter ASCT2 facilitation of glutamine uptake and mTORC1 kinase activation. Immunity.

[115] Marshall A.D., van Geldermalsen M., Otte N.J. (2017). ASCT2 regulates glutamine uptake and cell growth in endometrial carcinoma. Oncogenesis.

[116] Todorova V.K., Kaufmann Y., Luo S., Klimberg V.S. (2011). Tamoxifen and raloxifene suppress the proliferation of estrogen receptor-negative cells through inhibition of glutamine uptake. Cancer Chemother Pharmacol.

[117] Cheng T., Sudderth J., Yang C. (2011). Pyruvate carboxylase is required for glutamine-independent growth of tumor cells. Proc Natl Acad Sci U S A.

[118] Ai C., Sun X., Xiao S. (2023). CAFs targeted ultrasound-responsive nanodroplets loaded V9302 and GLULsiRNA to inhibit melanoma growth via glutamine metabolic reprogramming and tumor microenvironment remodeling. J Nanobiotechnol.

[119] Altman B.J., Stine Z.E., Dang C.V. (2016). From Krebs to clinic: glutamine metabolism to cancer therapy. Nat Rev Cancer.

[120] Oh M.H., Sun I.H., Zhao L. (2020). Targeting glutamine metabolism enhances tumor-specific immunity by modulating suppressive myeloid cells. J Clin Investig.

[121] Liu P.S., Chen Y.T., Li X. (2023). CD40 signal rewires fatty acid and glutamine metabolism for stimulating macrophage anti-tumorigenic functions. Nat Immunol.

[122] Wang Z., Li B., Li S. (2022). Metabolic control of CD47 expression through LAT2-mediated amino acid uptake promotes tumor immune evasion. Nat Commun.

[123] Zhao L., Rao X., Zheng R. (2023). Targeting glutamine metabolism with photodynamic immunotherapy for metastatic tumor eradication. J Contr Release.

[124] Chen G., Xu Q., Feng Z. (2022). Glutamine antagonist synergizes with electrodynamic therapy to induce tumor regression and systemic antitumor immunity. ACS Nano.

[125] Li Z., Lin J., Yin P. (2024). Ammonia death: a novel potential strategy to augment immunotherapy in cancer. Cancer Gene Ther.

[126] Rose C.F., Verkhratsky A., Parpura V. (2013). Astrocyte glutamine synthetase: pivotal in health and disease. Biochem Soc Trans.

[127] Häussinger D., Schliess F. (2007). Glutamine metabolism and signaling in the liver. Front Biosci.

[128] Zhao Y., Feng X., Chen Y. (2020). 5-fluorouracil enhances the antitumor activity of the glutaminase inhibitor CB-839 against *PIK3CA*-mutant colorectal cancers. Cancer Res.

[129] Pillai R., LeBoeuf S.E., Hao Y. (2024). Glutamine antagonist DRP-104 suppresses tumor growth and enhances response to checkpoint blockade in *KEAP1* mutant lung cancer. Sci Adv.

[130] Byrne K.T., Betts C.B., Mick R. (2021). Neoadjuvant selicrelumab, an agonist CD40 antibody, induces changes in the tumor microenvironment in patients with resectable pancreatic cancer. Clin Cancer Res.

[131] Ko A.H., Chao J., Noel M.S. (2025). A phase 2 study of sotigalimab, a CD40 agonist antibody, plus concurrent chemoradiation as neoadjuvant therapy for esophageal and gastroesophageal junction cancers. Cancer Res Commun.

[132] Weiss S.A., Sznol M., Shaheen M. (2024). A phase II trial of the CD40 agonistic antibody sotigalimab (APX005M) in combination with nivolumab in subjects with metastatic melanoma with confirmed disease progression on anti-PD-1 therapy. Clin Cancer Res.

[133] Luke J.J., Patel M.R., Blumenschein G.R. (2023). The PD-1- and LAG-3-targeting bispecific molecule tebotelimab in solid tumors and hematologic cancers: a phase 1 trial. Nat Med.

[134] Budczies J., Pfitzner B.M., Györffy B. (2015). Glutamate enrichment as new diagnostic opportunity in breast cancer. Int J Cancer.

[135] Liu Y., Zong X., Altea-Manzano P., Fu J. (2025). Amino acid metabolism in breast cancer: Pathogenic drivers and therapeutic opportunities. Protein Cell.

[136] Zhou L., Zhang Q., Zhu Q., Zhan Y., Li Y., Huang X. (2023). Role and therapeutic targeting of glutamine metabolism in non-small cell lung cancer. Oncol Lett.

[137] Wang X., Min S., Liu H. (2019). *Nf1* loss promotes *Kras*-driven lung adenocarcinoma and results in Psat1-mediated glutamate dependence. EMBO Mol Med.

[138] Romero R., Sayin V.I., Davidson S.M. (2017). Keap1 loss promotes Kras-driven lung cancer and results in dependence on glutaminolysis. Nat Med.

[139] Meijer T.W.H., Peeters W.J.M., Dubois L.J. (2018). Targeting glucose and glutamine metabolism combined with radiation therapy in non-small cell lung cancer. Lung Cancer.

[140] Yang W.H., Qiu Y., Stamatatos O., Janowitz T., Lukey M.J. (2021). Enhancing the efficacy of glutamine metabolism inhibitors in cancer therapy. Trends Cancer.

[141] Patel R., Alfarsi L.H., El-Ansari R. (2024). ATF4 as a prognostic marker and modulator of glutamine metabolism in oestrogen receptor-positive breast cancer. Pathobiology.

[142] Ren P., Yue M., Xiao D. (2015). ATF4 and N-Myc coordinate glutamine metabolism in MYCN-amplified neuroblastoma cells through ASCT2 activation. J Pathol.

[143] Forman A., Sotelo J. (2020). Tumor-based genetic testing and familial cancer risk. Cold Spring Harb Perspect Med.

[144] Neal J.T., Li X., Zhu J. (2018). Organoid modeling of the tumor immune microenvironment. Cell.

[145] Ekici S., Nye J.A., Neill S.G., Allen J.W., Shu H.K., Fleischer C.C. (2022). Glutamine imaging: a new avenue for glioma management. AJNR Am J Neuroradiol.

[146] Rajagopalan K.N., DeBerardinis R.J. (2011). Role of glutamine in cancer: therapeutic and imaging implications. J Nucl Med.

[147] Reis L.M.D., Adamoski D., Ornitz Oliveira Souza R. (2019). Dual inhibition of glutaminase and carnitine palmitoyltransferase decreases growth and migration of glutaminase inhibition-resistant triple-negative breast cancer cells. J Biol Chem.

[148] Jin J., Byun J.K., Choi Y.K., Park K.G. (2023). Targeting glutamine metabolism as a therapeutic strategy for cancer. Exp Mol Med.

[149] Méndez-Lucas A., Lin W., Driscoll P.C. (2020). Identifying strategies to target the metabolic flexibility of tumours. Nat Metab.

[150] Singleton D.C., Dechaume A.L., Murray P.M., Katt W.P., Baguley B.C., Leung E.Y. (2020). Pyruvate anaplerosis is a mechanism of resistance to pharmacological glutaminase inhibition in triple-receptor negative breast cancer. BMC Cancer.

[151] Biancur D.E., Paulo J.A., Małachowska B. (2017). Compensatory metabolic networks in pancreatic cancers upon perturbation of glutamine metabolism. Nat Commun.

[152] Polet F., Feron O. (2013). Endothelial cell metabolism and tumour angiogenesis: glucose and glutamine as essential fuels and lactate as the driving force. J Intern Med.

[153] Morrissey S.M., Zhang F., Ding C. (2021). Tumor-derived exosomes drive immunosuppressive macrophages in a pre-metastatic niche through glycolytic dominant metabolic reprogramming. Cell Metab.

[154] Byun J.K., Park M., Lee S. (2020). Inhibition of glutamine utilization synergizes with immune checkpoint inhibitor to promote antitumor immunity. Mol Cell.

